# Preoperative oral practices and incidence of postoperative complications in hospital medical-surgical procedures: A meta-analysis

**DOI:** 10.4317/medoral.25580

**Published:** 2023-04-07

**Authors:** Fabián Camus-Jansson, Natalia Longueira-Diaz, Barbara Salinas-Diaz, Abril Granic-Chinchón, Waldo Cueto-Urbina, Miguel Parra-Parra, Silvia Adriana Lopez-de-Blanc

**Affiliations:** 1 Department of Public Health, Dental School, Valparaíso University, Valparaíso, Chile; 2 Dental School, Valparaíso University, Valparaíso, Chile; 3 Department of Oral Pathology, National University of Cordoba, Cordoba, Argentina

## Abstract

**Background:**

Oral decay prior to a hospital medical-surgical procedure is a risk factor for the development of postoperative complications. However, perioperative oral practices as a protective factor have not been studied. This review aims to evaluate the effectiveness of perioperative oral practices in the reduction of risk of developing postoperative complications in in-hospital medical surgical procedures.

**Material and Methods:**

This review and meta-analysis was conducted according to Cochrane guidelines. Medline, Scopus, Scielo, and Cochrane were consulted. Articles of the previous 10 years concerning adult patients undergoing perioperative oral practices prior to hospital medical-surgical procedures, were included. Data of the type of perioperative oral practice, type of postoperative complication and measures of effect on the development of complications were extracted.

**Results:**

Of a pool of 1470 articles, 13 were included for systematic review and 10 for meta-analysis. The most common perioperative oral procedures were focalized approach (FA), referred to only the elimination of infectious foci in the oral cavity and comprehensive approach (CA), referred to a integral approach of the patient's oral health, both of which were mainly performed in oncologic surgeries, both were effective in the reduction of postoperative complications (RR=0.48, [95% CI 0.36 - 0.63]). The most reported postoperative complication was postoperative pneumonia.

**Conclusions:**

Perioperative oral management proved to be a protective factor against the development of postoperative complications.

** Key words:**General surgery, surgical oncology, perioperative care, clinical protocols, dental care, postoperative complications.

## Introduction

Postoperative complications are defined as deviations from the normal course of a surgery after the procedure and contribute significantly to patient morbidity, considerably increasing recovery time and hospital costs. These complications could not only affect the patient immediately after surgery, but also have late repercussions, even death (1). These vary according to the type of surgery and post-operative care; however, a considerable proportion of these are related to infection of compromised structures before, during and after surgery (2). Certain factors inherent to the critical condition of hospitalized patients must be considered, such as the presence of comorbidities, immunosuppression, the need for ventilatory support, the use of suction devices, feeding tubes, sedation, analgesia, and loss of protective reflexes as risk factors in the development of post operative complications (1,2). Since all surgical procedures that require the use of general anesthesia compromise the respiratory system through permeabilization of the airways, it is expected that pneumonia is the most frequent postoperative complication (3).

Evidence shows that oral decay prior to surgery is an important risk factor in the development of multiple kinds of postoperative complications (4-5), associated with the fact that oral cavity presents a great variety of pathogenic agents, with several studies showing that the different oral structures are colonized by different types of bacterial and fungal communities. Some of the post operative complications described associated with oral cavity bacteria are postoperative pneumonia (PN), infectious endocarditis (IE), surgical site infection (SSI), prosthetic joint infection (PJI) (3-7).

Perioperative oral management as a strategy to reduce the risk of oral bacteria colonization of structures has not been studied in depth as a protective factor prior to surgery to prevent the development of complications, as shown for example, by the fact that none of the predictive systems for postoperative risk consider the patient's oral health as a factor (3-4). It is also worth mentioning that there’s is no standardization even in surgeries where there is a relative consensus on the implementation of perioperative oral management as oncological surgery, thoracic surgery and prosthetic surgery, so that the techniques used depend on each clinician (3-8).

Therefore, the aim of this review is to evaluate, according to the literature, the effectiveness of perioperative oral management on the risk reduction of developing postoperative complications in medical surgical procedures, and to compare the effectiveness of different perioperative oral maneuvers in reducing systemic postoperative complications.

## Material and Methods

- Study Design

The present study is a systematic review and meta-analysis. Its structure is based on the PRISMA statement for Systematic Reviews and Meta-analysis and The Grading of Recommendations Assessment, Development and Evaluation (GRADE) approach (9-10).

- Research question

Our systematic review was conducted to answer the next question (designed using the PICO strategy): In patients undergoing hospital medical-surgical procedures, the application of preoperative oral practices reduces postoperative systemic complications?

Population (P): Patients undergoing hospital medical surgical procedures.

Intervention (I): Preoperative oral practices.

Control (C): Patients undergoing hospital medical surgical procedures not undergoing perioperative oral practices.

Outcome (O): Development of postoperative complications.

- Information search and strategy

To conduct this systematic review, four researchers independently used the MEDLINE, Scopus, Cochrane Library, and Scielo electronic databases. Articles published between 2012 and 2022 were included. The databases were searched between October and November 2022. MeSH (Medical Subject Headings) terms classified into patient, intervention, and outcome were used; in addition, the boolean operators "AND", "OR" and "NOT" were used (Table 1).

- Inclusion criteria

A. English, Spanish or Portuguese language.

B. Up to 10 years.

C. Randomized clinical trials, non-randomized clinical trials, and analytical observational studies.

D. Adults.

E. Studies using or evaluating perioperative oral procedures/maneuvers before in-hospital medical-surgical procedures.

- Exclusion Criteria

A. Cross-sectional and case-control studies.

B. Studies that consider the management of patients requiring mechanical ventilation without prior medical surgical procedure.

C. Studies that only include head and neck surgery.

D. Studies that only include hygiene maneuvers like toothbrushing and mouthwashes or non-dental professional intervention.

E. Patients receiving oral care following surgery that includes procedures in addition to routine tooth brushing.

F. Studies that do not specify which perioperative oral maneuvers were performed.

G. Studies in which both groups underwent the same perioperative oral practices.

**Table 1:**
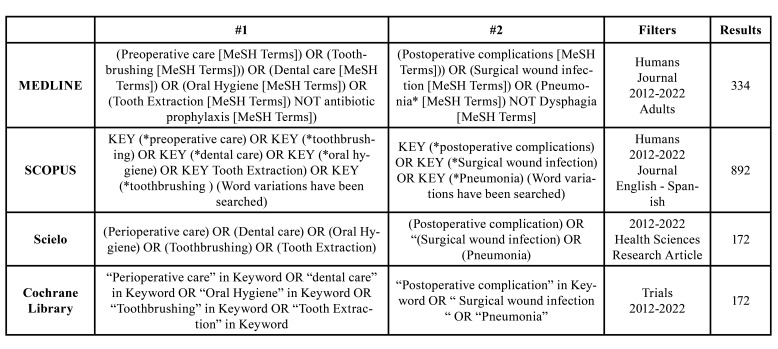
Search strategy.

- Study selection

The researchers individually looked for articles in the different electronic databases, using combinations of the MeSH terms. The articles encountered were tabulated using the Google Spreadsheet and managed through the Mendeley 2.80.1.

- Data extraction

Four reviewers independently evaluated each of the titles and abstracts of the articles found according to the following steps: non-relevant articles were excluded; then the full texts were analyzed, discarding those that did not meet the inclusion and exclusion criteria. Any discrepancies between the investigators were resolved through further analysis and discussion.

- Quality assessment

The studies resulting from the search were independently reviewed, and the risk of bias in the included studies was assessed. Data from studies with similar interventions and outcomes were grouped.

For the quality assessment, which focused on detecting the main sources of bias, it was necessary to establish a standardized approach prior to the assessment. Discrepancies were settled through discussion and consensus. For this analysis, Newcastle Ottawa scale (NCO) for nonrandomized studies was used.

- Outcome Measures

The following study variables were identified: 

1. Place where the study was conducted.

2.The number of selected groups and participants.

3. Perioperative oral maneuvers performed.

4. Medical-surgical procedure performed.

5. Type of postoperative complications developed.

6. Measures of effect on the development of postoperative complications.

- Statistical Analysis

The measures of effects were considered according to study design and type of variable. In this case, Relative Risk (RR) was used, under a 95% confidence interval (CI) using the random-effects model to incorporate heterogeneity. A *p-value* of <0.05 was considered statistically significant. Heterogeneity among studies was assessed using the I2 statistical test and the X2 test with a value of <0.05. The studies and analyses were subsequently presented in forest plots, subdivided in type of perioperative oral practice. The RevMan tool (Review Management 5.4) was employed.

## Results

- Results of the study selection process

**Figure 1:**
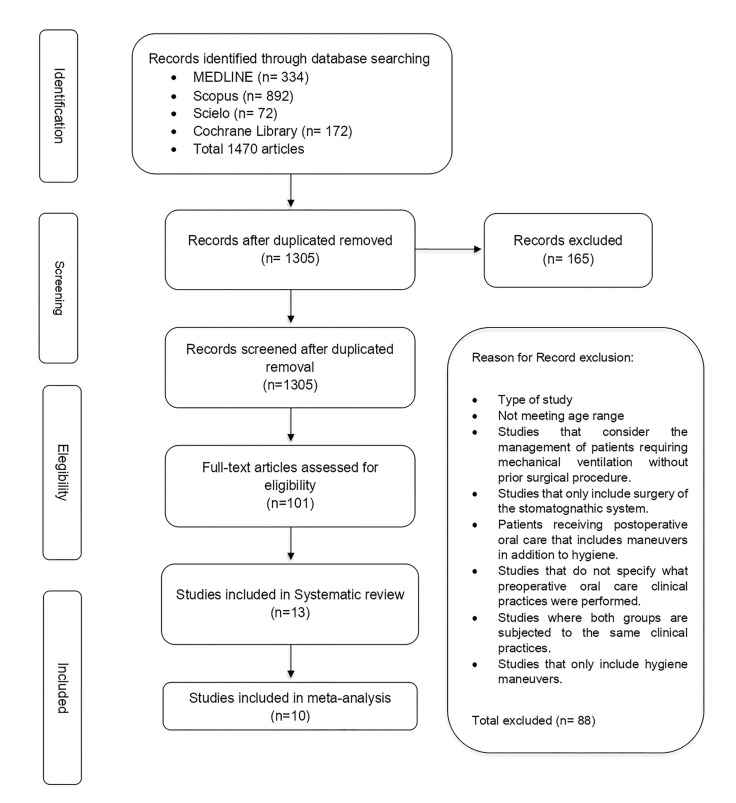
Record flow diagram.

A total of 1470 articles were identified, of which 165 duplicate articles were discarded, yielding a total of 1305 studies remaining. Subsequently, the analysis of the titles and abstracts of the resulting studies yielded a total of 101 articles after applying the inclusion criteria. Then, exclusion criteria were applied, finally obtaining 13 articles included in the systematic review, and of these, 10 were included in the meta-analysis (Fig. 1) (11-23).

The reasons for the exclusion of articles from the systematic review were mainly divided into: 

1. Oral post-operative management was performed [3].

2. The study subjects did not undergo surgery, but only assisted ventilation [16].

3. The format of the perioperative oral care procedures is not specified [3].

4. Only surgeries involving the head and neck were considered [15].

5. The study design does not comply with what was considered for the selection of the articles [2].

6. Studies in which both groups underwent the same perioperative oral practices [2].

7. Studies only included hygiene maneuvers or non-dental professional intervention [47].

Reasons for exclusion of articles from the meta-analysis.

1. The studies compared two protocols of perioperative oral practices, without a control group [3].

- Methodological Quality

The Newcastle Ottawa scale was used. Of the 13 studies considered, 5 had a high risk of bias (11-13,20-21) (Jia *et al*, Hasegawa *et al*, Sato *et al*, Soutome *et al*, Konstanty *et al*), and 8 studies low risk of bias (14-19,22-23)(Nobuhara *et al*, Rao *et al*, Yamada *et al*, Kurasawa *et al*, Ishikawa *et al*, Iwata *et al*, Nobuhara *et al*, Sonn *et al*) (Table 2).

- Perioperative dental practices

Two types of maneuvers were identified: focalized approach (FA) (11,13-19,21-23) and comprehensive approach (CA) (12,18,20), although one study performed incomplete CA (12).

FA could comprise just only exodontia (16,18) or more procedures like diagnostic examination, extraction of infected teeth, prophylaxis, and professional periodontal treatment, in addition to self-care instructions (11,13-17,19,21-23). In some cases, tongue cleaning (14-15,17,23) and prosthesis cleaning (14-23). Studies that applied CA protocols included: diagnostic examination, restoration of decayed teeth, extraction of teeth with poor prognosis, and complete periodontal treatment (including root planing), and root canal treatment (12,18).

Among the perioperative oral care practices that were compared in the studies, 10 evaluated the effectiveness of FA, versus no intervention (11,13-17,19,21-23), 1 study evaluated CA versus no intervention (20), 1 study evaluated CA versus FA18 and 1 study evaluated CA versus incomplete treatment (12).

While some studies did not specify the time between the performance of the perioperative care and surgery (15,17-19), others only specified that it began during the hospitalization process and ended before surgery (12,13).

**Table 2:**
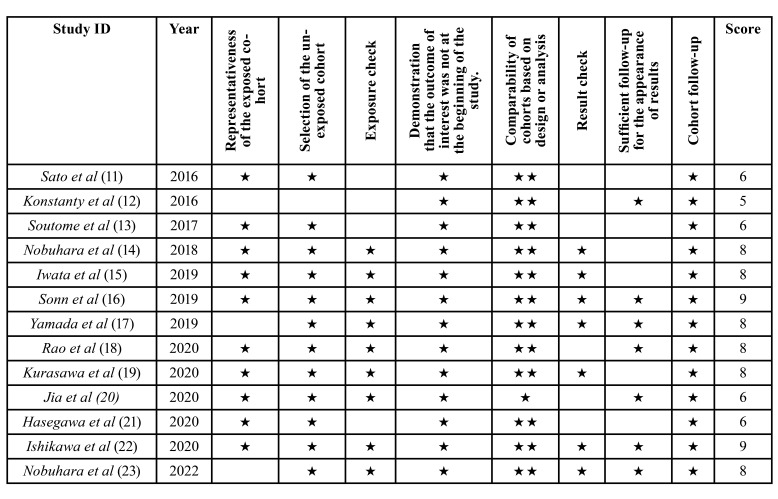
Risk of bias assessment for the non-randomized articles included in this review according to the Newcastle Ottawa scale.

Other studies were more specific, stating concrete time intervals, such as Sonn *et al* (52 days average before surgery) (16), Jia *et al* (5 days before surgery) (20), Hasegawa *et al* (2 weeks prior) (21), and Sato *et al* (1 week before surgery) (11), Ishikawa *et al* (1-4 days before surgery) (15), Nobuhara *et al* (2-10 days before surgery) (23).

Post-surgery care indications included tooth brushing, flossing, interdental cleaning (with interdental brushes or water pick), and rinsing with water (13-14,17,20-23); some studies included cleaning of removable prosthesis (14,17,21). Other studies did not specify indications (11,12,15-16,18-19,22). No study considered the long-term effect of the application of hygiene control measures after surgery.

The studies that evaluated FA versus no intervention obtained positive results, concluding that perioperative oral care such as this corresponds to an indication that is effective in reducing the development of postoperative complications, which was evaluated through measures of association (11,13-15,17-19,21,23), except Sonn *et al* and Ishikawa *et al*, that didn’t find significantly differences between FA groups versus no intervention group (16,22).

In the study where CA was evaluated against no intervention, it was concluded that integral treatment is an effective indication in reducing the incidence of postoperative complications (18) (Table 3).

**Table 3:**
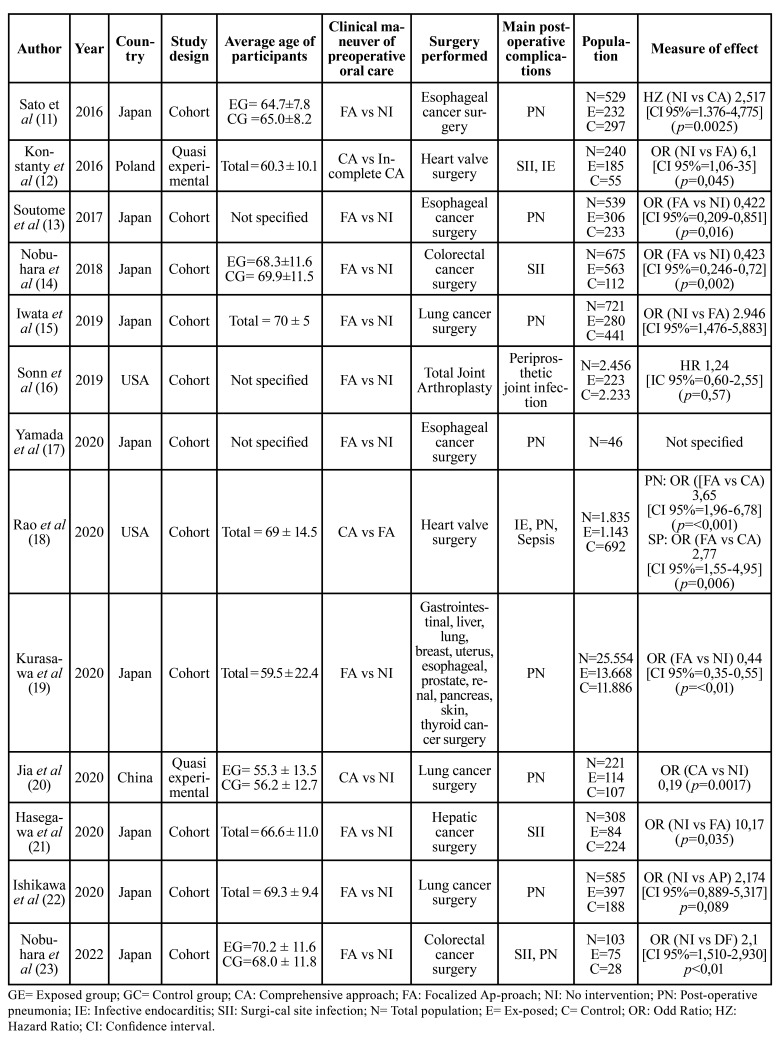
Review and results of studies included.

- Comparation between FA and CA

FA and CA showed statistically significant results as protective factors against the development of postoperative complications in most studies (11-15,17-23). Regarding the relevant differences between both protocols, CA considers a greater number of procedures, as well as an integral approach of the oral health of patients, considering definitive restorations such as composites and crowns. On the other hand, FA only considers control of infection foci, such as extractions, periodontal treatment and hygiene, procedures that also are considered in CA. Only Rao *et al* (18) compared CA and FA, and obtained results according to the type of postoperative complication: the patients of the FA group developed less PN (OR= 3.65; [95% CI= 1.96-6.78 ]; *p*<0.01), while the patients of the CA group developed less sepsis (OR=2.77; [95% CI=1.55-4.95]; *p*=0.006), while there were no statistically significant differences in the development of IE (OR= 2.49, [95% CI= 0.70-8.86], *p*=0.159). Another study that modified two protocols corresponds to Kontansty *et al* (12), which compared CA vs. incomplete CA, without specifying what maneuvers were not completed in the procedures. Complete AC showed a lower risk in the development of postoperative complications compared to incomplete AC (OR= 6.1; [95% CI = 1.06-35.00]; *p*=0.042).

- Development of postoperative complications

The total sample among the 13 studies included 34.946 patients, of whom 1010 (2.89%) developed some form of complication after surgery. Of these, 17.791 underwent perioperative oral interventions, while 17.042 did not undergo any. Of the patients who underwent perioperative oral management, 407 developed complications, while in the unexposed group 603 did.

Postoperative pneumonia was the most reported complication, being reported in 9 of the 13 studies. Diagnostic criteria were chest radiographic opacity, fever, leukocytosis, or leukopenia, and purulent sputum (11,13,15,17-20,22-23). Only one study specified severity criteria, as in the case of Sato *et al* (11), who diagnosed according to Clavien-Dindo Classification. Three studies did not specify their diagnostic criteria (18-20). Postoperative pneumonia was reported in patients undergoing oncologic surgery of different structures, mainly of the thoracic structures (11,13,15,17,20,22), in some cases also heart surgery (18), abdominal oncologic surgery and structures of other systems (19,23).

The second most reported complication in the literature was surgical site infection (SSI). Diagnostic criteria were purulent discharge remaining from the operative wound, and the presence of culture-positive bacteria (12,14,21,23). The surgeries that reported the occurrence of this complication were colorectal oncologic surgeries (14,21,23) and heart valve surgeries (12).

Only two studies reported infective endocarditis as a postoperative complication, both being associated with heart and valve surgery (12,18). The following criteria were used for diagnosis: infection (confirmed by the presence of fever > 38°C), wheezing, and leukocytosis (12).

Other complications were reported: 1 study reported prosthetic joint infection in prosthetic surgery and its diagnosis criteria was based on Musculoskeletal Infection Society criteria (16) and one study reported sepsis as postoperative complication in cardiac surgery (18), but its diagnostic criteria were not specified.

- Quantitative analysis of the data (meta-analysis)

Perioperative dental practices on the incidence of postoperative complications

**Figure 2:**
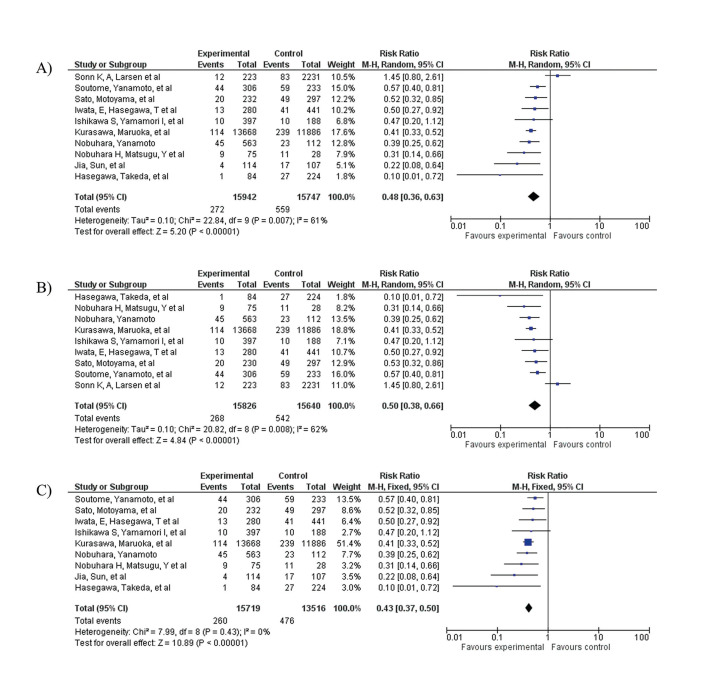
A) Forest plot: Preoperative oral procedures and postoperative complications. B) Forest plot: Focused approach and postoperative complications meta-analysis. C) Forest plot: Preoperative oral procedures and postoperative complications in oncological surgery meta-analysis.

Random effect was used. The studies that crossed the line of no effect was Sonn *et al* and Ishikawa *et al* (16,22). The intervention favors the exposed, showing perioperative oral practices as a protective factor (RR=0.48, [95% CI 0.36 - 0.63]). Significant heterogeneity (*p*=0.007) with inconsistency coefficient of I² = 61% (Fig. 2).

**Figure 3:**
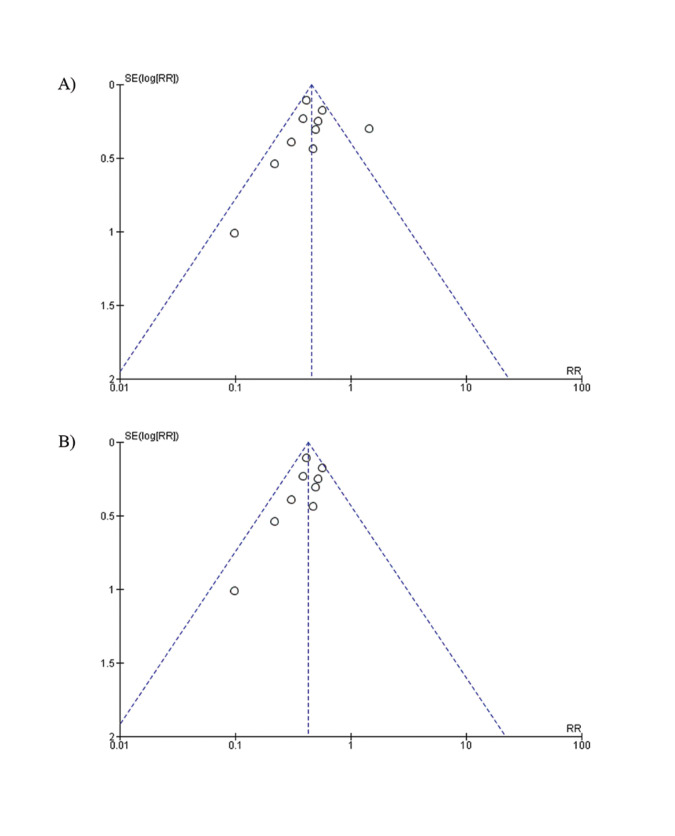
A) Funnel plot: Preoperative oral procedures and postoperative complications. B) Funnel plot: Preoperative oral procedures and postoperative complications in oncological surgery..

Not considerably asymmetry appeared in the Funnel plot, revealing homogeneity across the studies. No publication bias was observed, due to the symmetry of the dispersion, but this may be due to the small number of articles examined. The standard error of the studies was low, with most of them being central and close to the tip of the triangle. (Fig. 3).

- FA on the incidence of postoperative complications

Random effect was used. No study crossed the line of no-effect. It was observed that the intervention favored the exposed, showing the removal of all sources of active oral infection as a protective factor (RR=0.50, [95% CI 0.38 - 0.66]). Significant heterogeneity (*p*=0.008) with inconsistency coefficient of I² = 62% (Fig. 2).

- Perioperative dental practices on the incidence of postoperative complications in oncologic surgery

Fixed effect was used. The only study that crossed the line of no-effect was Ishikawa *et al* (22). It was observed that the intervention favored the exposed, showing the removal of all sources of active oral infection as a protective factor (RR=0.43, [95% CI 0.37 - 0.50]). Not significant heterogeneity (*p*=0.43) with inconsistency coefficient of I² = 0%. (Fig. 3).

Not considerably asymmetry appeared in the Funnel plot, revealing homogeneity across the studies. No publication bias was observed, due to the symmetry of the dispersion, but this may be due to the small number of articles examined. The standard error of the studies was low, with most of them being central and close to the tip of the triangle. (Fig. 3).

- Level of evidence analysis

This review included mostly observational studies in its analysis, which means that it starts from a low level of evidence. In the risk assessment using NCO, the studies with the highest weight achieved a high score, implying a low risk of bias. The confidence intervals of the pooled association measure of the studies in their quantitative analysis were consistently shown to be on the benefit side. The studies showed heterogeneity among them. The confidence intervals for the measures of effect were narrow. It was not possible detect serious publication bias.

Quantitative analysis also showed a strong association between decreased risk of developing complications and the application of perioperative oral practices (RR=0.48, [95% CI 0.36 - 0.63]). On the other hand, the studies controlled confounding variables through statistical methods; however, no study analyzed the dose-response gradient or the long-term effect of the application of perioperative oral practices.

## Discussion

In this systematic review and meta-analysis, the results of 13 studies conducted in China, Japan, USA, and Poland (11-23) were analyzed, which sought to test the effect of perioperative oral practices (CA and FA) on the incidence of postoperative complications in patients undergoing surgery.

The quantitative analysis showed that perioperative oral practices are a clinically significant protective factor against the development of postoperative complications (RR=0.48, [95% CI 0.36 - 0.63]) such as pneumonia, surgical site infection, and endocarditis. This could be explained by the fact that the oral cavity is a reservoir of microorganisms that can cause infection in adjacent or remote organs. Four biological mechanisms could induce the appearance of infectious conditions associated with oral microbiota: direct transfer of oral bacteria, which may be implicated in upper respiratory tract infections and SSI in head and neck oncologic surgeries; intravascular invasion of bacteria, which is transferred to the blood and lymphatic vessels, colonizing remote structures; the passage of endotoxins through blood and lymphatic vessels; and the direct ingestion of oral pathogens that can alter the intestinal microbiota (5-6), the first two being the most relevant in the development of postoperative complications.

Poor oral hygiene can lead to periodontitis, a chronic inflammatory disease characterized by the presence of a reservoir of complex microbiological communities. Periodontal pockets can serve as a reservoir of potential pathogens for respiratory tract infections, which can penetrate the adjacent microvasculature and lead to bacteremia. Therefore, by performing perioperative clinical oral care, the microbiological load is reduced, controlling bacteremia, and therefore reducing the risk of postoperative complications (5,24).

Nosocomial infections are one of the most important contributors to patient morbidity and mortality, significantly increasing hospitalization time and total costs. As an example, deep sternal infection (a type of SSI) in open-heart surgery in Denmark has an estimated cost per treatment of €40,000; hence, it is in this context that perioperative oral care practices take on special clinical and practical importance (25). For this reason, the importance of the elimination of infectious foci before major surgeries has been previously investigated (5,26-27), which can be traced back to classical considerations, where well-established protocols detail the elimination of infectious foci as a requirement prior to the start of some surgeries or invasive therapies (28). For example, it has been reported that, in patients undergoing elective spinal surgery, 47% of patients who develop SSI suffer from some degree of periodontitis or that in patients undergoing liver transplantation, poor oral health is an important factor in the development of postoperative complications (29-30), with more studies reaching similar conclusions, regarding different types of procedures (5,24,26,31-33).

Pneumonia was the most reported complication in oncologic and heart surgery, this has been associated with various factors, such as the level of immune compromise, the transfer of bacteria from the oral cavity and adjacent structures to the upper airway through the implementation of assisted ventilation, and poor oral condition (34). Several studies have reported that patients with poor oral hygiene have significantly higher rates of pneumonia (6-26).

Similarly, research has been conducted to determine which perioperative oral practices are necessary prior to mechanical intubation of patients. This is complex in emergency procedures, such as immediate intubation after severe complications associated with acute respiratory conditions, where it is impossible to carry out the planned oral procedures described in the present study. This takes on importance in the current context of the COVID-19 pandemia, where patients often require immediate mechanical ventilation (27,35).

The second most reported complication corresponded to SSI (12,14,21,23), in which the described mechanism corresponds to the transition of oral bacteria through blood and lymphatic pathways, leading to colonization of the surgical site. This complication is common in gastrointestinal or liver surgeries, so the use of minimally invasive methods, such as laparoscopic surgery, has become an attractive option (30,36).

When comparing perioperative oral practices versus no action, these proved to be an effective maneuver in reducing the development of postoperative complications (11,13-17,19,20-21,23). The perioperative oral care protocol with the greatest impact in the literature corresponded to FA, which is not only supported by the literature presented in this review (11,13-15,17-19,21,23), and meta-analysis (RR=0.50, [95% CI= 0.38 - 0.63]), but also by its incorporation as a policy in the Japanese public system (19). It is important to emphasize this point, since patients undergoing CA are exposed to greater morbidity, greater private or fiscal expenditure in their approach, and extensive time requirements for their implementation (16), so when evaluating the clinical significance of each of the protocols, it is important to consider whether it is convenient to apply more complex practices with similar results.

The surgery with the greatest evidence and effectiveness of the perioperative oral procedures in reduction of postoperative complications corresponded to oncological surgery (RR=0.43, [95% CI= 0.37-0.50]). Several studies have reported poor oral health as a risk factor in the development of postoperative complications in oncological surgery for the treatment of tumors that compromise the oral cavity, larynx, oropharynx, and esophagus (4,26). Elimination of infectious foci is necessary prior to head and neck oncological surgery in established protocols (8), but evidence is limited on surgeries of other structures. This review shows the importance of oral health in the post-surgical evolution of patients undergoing oncological surgeries of gastrointestinal and thoracic structures.

The evaluation of bias in observational and quasi-experimental analytical studies is complex to perform because there are a series of variables that the researcher does not handle, and tools used in experimental designs to evaluate them are difficult to apply; Therefore, the Newcastle Ottawa scale was used because it is the most commonly used for cohort designs and provides an approximation of the biases of each study, according to the score in the scale. In the bias analysis, the studies with the highest significance showed a low risk of bias. When evaluating quality of the evidence (based on the criteria proposed by GRADE (10), given the study design and the effective fulfillment of GRADE criteria such as consistency, precision, certainty, and low risk of publication bias, added to the adequate management of confounding factors, the level of evidence should at least be considered low. Evidence suggests that perioperative oral practices reduce postoperative complications in medical-surgical procedures, but more randomized clinical studies are needed in order to obtain conclusive results.

The findings in the present study could be extrapolated to those described by Pedersen *et al* (25) in a systematic review on the effectiveness of different clinical practices of oral-dental care in the reduction of postoperative complications in adult thoracic surgery, where mainly antibiotic and hygiene protocols were used, in which similar results were obtained (RR=0.48 [95% CI=0.27-0.84]), concluding that perioperative oral management corresponds to a protective factor against the development of complications. However, other studies have concluded that in certain types of surgeries the evidence does not support the importance of perioperative clinical practices in the development of complications. Lockhart *et al* (31), in an extensive review, which evaluated the effectiveness of perioperative dental management in reducing complications in heart valve surgery concerning the development of IE (RR=1.01 [95% CI=0.76-1.33]), concluded that there were no significant differences in the reduction of complications in patients on whom perioperative oral practices were performed. It should be noted that in the quantitative analysis, only three studies were included. It should be considered that in our review there are only few studies where cardiac surgery has been performed.

Another type of surgery where the evidence is inconclusive corresponds to prosthetic surgeries, as shown by Barrere *et al* (37) in their systematic review, where they conclude that there is no substantial evidence to support that there is a reduction in postoperative complications associated with prosthetic surgeries, even suggesting that preoperative oral maneuvers could have an overestimated effect in reducing postoperative complications. In this case, the authors emphasize that the evidence they had was extremely heterogeneous, and that none of the research they analyzed really answered their research question. Other factors that should be considered is that a considerable part of their methodological designs correspond to case series (37). These results contrast with those of our review, considering that the only study that performed preoperative oral maneuvers in patients undergoing prosthetic surgery obtained no statistically significant results, in contrast to oncological surgeries, where the evidence of our review convincingly shows the importance of perioperative oral care in reduction of the risk of developing postoperative complications.

A limitation of our review is that most of the studies were carried out in Japan. Moreover, certain authors tend to show up repeatedly within the authors of the studies such as Hasegawa T and Nobuhara H (14,21,23). We evaluate a possible overlapping of sample, causing an overrepresentation of the effect, but the studies include patients with different pathologies or different hospitals. Therefore, the sample units are not repeated between one study and the other.

A possible explanation for the large number of Japanese studies is that in Japan, universal care was implemented in 1961, and from that moment on, the country’s health indicators have gradually improved, and consequently, the government has placed the focus on increasing the number of human resources in health, as well as investing in training for medical and health personnel. Concerning oral health, since 1989, education and prevention activities have steadily increased, and as a result, there has been an improvement in the Japanese population's awareness of the importance of oral health (38). In 2012, Perioperative Oral Management (POM) was introduced into the Japanese universal health insurance system to prevent postoperative complications in cancer patients undergoing surgery. This refers to out-of-hospital oral care provided by a dentist prior to in-hospital medical-surgical treatment, which includes a complete dental evaluation and the corresponding dental treatment in cases of high risk of infection (13). The purpose of this is to ensure adequate oral health conditions at the time of surgery. Due to the importance given to public dental policies in Japan, a considerable number of the studies reviewed in the present meta-analysis are from this country, considering that the access to the hospitals, records and patients is easier, promoting the investigation in this area. Taking this into account, this review reveals the need to carry out further research in this area, unifying methodologies that will allow comparisons between different countries, not only Asian ones. Studies should also be carried out to compare FA and CA techniques, as well as to identify and study other protocols. In addition, a comparison could be made between the use of mouthwashes or antiseptics such as chlorhexidine with perioperative oral management, although they are different in concept, considering that there is ample evidence about its effectiveness in the reduction of postoperative complications as a hygiene procedure (6,39-40). It is also advisable to carry out an economic analysis to evaluate the cost-benefit of implementing these protocols in public and private services.

In conclusion, the rating of the evidence collected is low because of the types of designs. However, the evidence suggests that perioperative oral care practices, in particular FA reduce postoperative complications after medical surgical procedures, particularly in oncological surgical procedures, the perioperative oral maneuver with more evidence was FA. Therefore, it is important to evaluate the implementation of perioperative clinical protocols as a public policy to reduce the risk of postoperative complications in patients who will undergo medical-surgical procedures.

## References

[1] Aahlin EK, Olsen F, Uleberg B, Jacobsen BK, Lassen K (2016). Major postoperative complications are associated with impaired long-term survival after gastro-esophageal and pancreatic cancer surgery: A complete national cohort study. BMC Surg.

[2] Ou L, Chen J, Hillman K, Flabouris A, Parr M, Assareh H (2017). The impact of post-operative sepsis on mortality after hospital discharge among elective surgical patients: a population-based cohort study. Crit Care.

[3] Inai Y, Nomura Y, Takarada T, Hanada N, Wada N (2020). Risk factors for postoperative pneumonia according to examination findings before surgery under general anesthesia. Clin Oral Investig.

[4] Zhang Y, Wang X, Li H, Ni C, Du Z, Yan F (2018). Human oral microbiota and its modulation for oral health. Biomed Pharmacother.

[5] Dhotre S, Jahagirdar V, Suryawanshi N, Davane M, Patil R, Nagoba B (2018). Assessment of periodontitis and its role in viridans streptococcal bacteremia and infective endocarditis. Indian Heart J.

[6] Zhao T, Wu X, Zhang Q, Li C, Worthington HV, Hua F (2020). Oral hygiene care for critically ill patients to prevent ventilator-associated pneumonia. Cochrane Database Syst Rev.

[7] Baddour LM, Wilson WR, Bayer AS, Fowler VG, Bolger AF, Levison ME (2005). Infective endocarditis: diagnosis, antimicrobial therapy, and management of complications: a statement for healthcare professionals from the Committee on Rheumatic Fever, Endocarditis, and Kawasaki Disease, Council on Cardiovascular Disease in the Young, and the Councils on Clinical Cardiology, Stroke, and Cardiovascular Surgery and Anesthesia, American Heart Association: endorsed by the Infectious Diseases Society of America. Circulation.

[8] PDQ Supportive and Palliative Care Editorial Board (2022). Oral Complications of Chemotherapy and Head/Neck Radiation (PDQ®): Health Professional Version. In: PDQ Cancer Information Summaries. Bethesda.

[9] Liberati A, Altman DG, Tetzlaff J, Mulrow C, Gøtzsche PC, Ioannidis JPA (2009). The PRISMA Statement for Reporting Systematic Reviews and Meta-Analyses of Studies That Evaluate Health Care Interventions: Explanation and Elaboration. PLoS Med.

[10] Aguayo-Albasini JL, Flores-Pastor B, Soria-Aledo V (2014). [GRADE system: classification of quality of evidence and strength of recommendation]. Cir Esp.

[11] Sato Y, Motoyama S, Takano H, Nakata A, Liu J, Harimaya D (2016). Esophageal Cancer Patients Have a High Incidence of Severe Periodontitis and Preoperative Dental Care Reduces the Likelihood of Severe Pneumonia after Esophagectomy. Dig Surg.

[12] Konstanty-Kalandyk J, Kalandyk-Konstanty A, Kapelak B, Zarzecka J, Drwila R, Kieltyka A (2016). Incomplete oral sanation as a risk factor for elevated leucocytosis and postoperative infection. Kardiol Pol.

[13] Soutome S, Yanamoto S, Funahara M, Hasegawa T, Komori T, Yamada SI (2017). Effect of perioperative oral care on prevention of postoperative pneumonia associated with esophageal cancer surgery. Medicine (Baltimore).

[14] Nobuhara H, Yanamoto S, Funahara M, Matsugu Y, Hayashida S, Soutome S (2018). Effect of perioperative oral management on the prevention of surgical site infection after colorectal cancer surgery: A multicenter retrospective analysis of 698 patients via analysis of covariance using propensity score. Medicine (Baltimore).

[15] Iwata E, Hasegawa T, Yamada SI, Kawashita Y, Yoshimatsu M, Mizutani T (2019). Effects of perioperative oral care on prevention of postoperative pneumonia after lung resection: Multicenter retrospective study with propensity score matching analysis. Surgery.

[16] Sonn KA, Larsen CG, Adams W, Brown NM, McAsey CJ (2019). Effect of Preoperative Dental Extraction on Postoperative Complications After Total Joint Arthroplasty. J Arthroplasty.

[17] Yamada Y, Yurikusa T, Furukawa K, Tsubosa Y, Niihara M, Mori K (2019). The Effect of Improving Oral Hygiene through Professional Oral Care to Reduce the Incidence of Pneumonia Post-esophagectomy in Esophageal Cancer. Keio J Med.

[18] Rao NR, Treister N, Axtell A, Muhlbauer J, He P, Lau A (2020). Preoperative dental screening prior to cardiac valve surgery and 90-day postoperative mortality. J Card Surg.

[19] Kurasawa Y, Maruoka Y, Sekiya H, Negishi A, Mukohyama H, Shigematsu S (2020). Pneumonia prevention effects of perioperative oral management in approximately 25,000 patients following cancer surgery. Clin Exp Dent Res.

[20] Jia C, Sun M, Wang W, Li C, Li X, Zhang X (2020). Effect of oral plaque control on postoperative pneumonia following lung cancer surgery. Thorac Cancer.

[21] Hasegawa T, Takeda D, Tanaka M, Amano R, Saito I, Kakei Y (2021). Effects of preoperative dental examination and oral hygiene instruction on surgical site infection after hepatectomy: a retrospective study. Support Care Cancer.

[22] Ishikawa S, Yamamori I, Takamori S, Kitabatake K, Edamatsu K, Sugano A (2021). Evaluation of effects of perioperative oral care intervention on hospitalization stay and postoperative infection in patients undergoing lung cancer intervention. Support Care Cancer.

[23] Nobuhara H, Matsugu Y, Soutome S, Hayashida S, Hasegawa T, Akashi M (2022). Perioperative oral care can prevent surgical site infection after colorectal cancer surgery: A multicenter, retrospective study of 1,926 cases analyzed by propensity score matching. Surgery.

[24] Emery DC, Cerajewska TL, Seong J, Davies M, Paterson A, Allen-Birt SJ (2021). Comparison of Blood Bacterial Communities in Periodontal Health and Periodontal Disease. Front Cell Infect Microbiol.

[25] Pedersen PU, Larsen P, Håkonsen SJ (2016). The effectiveness of systematic perioperative oral hygiene in reduction of postoperative respiratory tract infections after elective thoracic surgery in adults: a systematic review. JBI Database System Rev Implement Rep.

[26] Ishimaru M, Ono S, Matsui H, Yasunaga H (2019). Association between perioperative oral care and postoperative pneumonia after cancer resection: conventional versus high-dimensional propensity score matching analysis. Clin Oral Investig.

[27] Kishimoto Y, Sogami T, Uozumi R, Funakoshi M, Miyamoto SI, Kitamura M (2018). Complications After Endoscopic Laryngopharyngeal Surgery. Laryngoscope.

[28] Mangram AJ, Horan TC, Pearson ML, Silver LC, Jarvis WR (1999). Guideline for Prevention of Surgical Site Infection, 1999. Centers for Disease Control and Prevention (CDC) Hospital Infection Control Practices Advisory Committee. Am J Infect Control.

[29] Mirzashahi B, Tonkaboni A, Chehrassan M, Doosti R, Kharazifard MJ (2019). The role of poor oral health in surgical site infection following elective spinal surgery. Musculoskelet Surg.

[30] Kauffels A, Schmalz G, Kollmar O, Slotta JE, Weig M, Groß U (2017). Oral findings and dental behaviour before and after liver transplantation - a single-centre cross-sectional study. Int Dent J.

[31] Lockhart PB, DeLong HR, Lipman RD, Abt E, Baddour LM, Colvin M (2019). Effect of dental treatment before cardiac valve surgery: Systematic review and meta-analysis. J Am Dent Assoc.

[32] Tokarski AT, Patel RG, Parvizi J, Deirmengian GK (2014). Dental clearance prior to elective arthroplasty may not be needed for everyone. J Arthroplasty.

[33] Devi S, Singh N (2014). Dental care during and after radiotherapy in head and neck cancer. Natl J Maxillofac Surg.

[34] Messika J, La Combe B, Ricard JD (2018). Oropharyngeal colonization: epidemiology, treatment and ventilator-associated pneumonia prevention. Ann Transl Med.

[35] Hur K, Price CPE, Gray EL, Gulati RK, Maksimoski M, Racette SD (2020). Factors Associated With Intubation and Prolonged Intubation in Hospitalized Patients With COVID-19. Otolaryngol Head Neck Surg.

[36] Tan S, Wu G, Zhuang Q, Xi Q, Meng Q, Jiang Y (2016). Laparoscopic versus open repair for perforated peptic ulcer: A meta-analysis of randomized controlled trials. Int J Surg.

[37] Barrere S, Reina N, Peters OA, Rapp L, Vergnes JN, Maret D (2019). Dental assessment prior to orthopedic surgery: A systematic review. Orthop Traumatol Surg Res.

[38] Akashi H, Osanai Y, Akashi R (2015). Human resources for health development: toward realizing Universal Health Coverage in Japan. Biosci Trends.

[39] Hua F, Xie H, Worthington HV, Furness S, Zhang Q, Li C (2016). Oral hygiene care for critically ill patients to prevent ventilator-associated pneumonia. Cochrane Database Syst Rev.

[40] Bardia A, Blitz D, Dai F, Hersey D, Jinadasa S, Tickoo M (2019). Preoperative chlorhexidine mouthwash to reduce pneumonia after cardiac surgery: A systematic review and meta-analysis. J Thorac Cardiovasc Surg.

